# The Lateral Compressive Buckling Performance of Aluminum Honeycomb Panels for Long-Span Hollow Core Roofs

**DOI:** 10.3390/ma9060444

**Published:** 2016-06-03

**Authors:** Caiqi Zhao, Weidong Zheng, Jun Ma, Yangjian Zhao

**Affiliations:** Key Laboratory of Concrete and Prestressed Concrete Structure, Ministry of Education, School of Civil Engineering, Southeast University, Nanjing 210096, China; zwdlyj@163.com (W.Z.); majunmajunb@163.com (J.M.); zyj66720200@163.com (Y.Z.)

**Keywords:** long-span hollow core roof, bionic structure, honeycomb panel structural system, lateral compressive test, nonlinear buckling analysis, critical lateral compressive load

## Abstract

To solve the problem of critical buckling in the structural analysis and design of the new long-span hollow core roof architecture proposed in this paper (referred to as a “honeycomb panel structural system” (HSSS)), lateral compression tests and finite element analyses were employed in this study to examine the lateral compressive buckling performance of this new type of honeycomb panel with different length-to-thickness ratios. The results led to two main conclusions: (1) Under the experimental conditions that were used, honeycomb panels with the same planar dimensions but different thicknesses had the same compressive stiffness immediately before buckling, while the lateral compressive buckling load-bearing capacity initially increased rapidly with an increasing honeycomb core thickness and then approached the same limiting value; (2) The compressive stiffnesses of test pieces with the same thickness but different lengths were different, while the maximum lateral compressive buckling loads were very similar. Overall instability failure is prone to occur in long and flexible honeycomb panels. In addition, the errors between the lateral compressive buckling loads from the experiment and the finite element simulations are within 6%, which demonstrates the effectiveness of the nonlinear finite element analysis and provides a theoretical basis for future analysis and design for this new type of spatial structure.

## 1. Introduction

**Figure 1:**
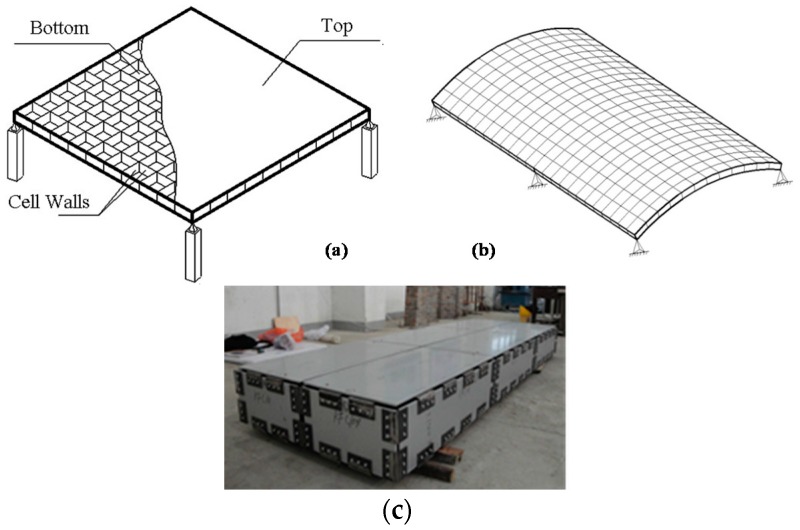
Typical long-span hollow core roofs formed by honeycomb panels: (**a**) flat type; (**b**) curved type; (**c**) assembly unit.

A honeycomb panel, as the name suggests, is a man-made structure that is inspired by natural honeycombs and is a typical type of high-strength lightweight bionic structure [1,2,3,4]. Chen *et al.* reported that only the forewings of beetles are fully integrated honeycomb panels and noted that the honeycomb itself is only the core structure of honeycomb panels and not a panel structure [5,6,7,8]. Compared to core-only honeycomb panels in nature, bionic integrated honeycomb panels have the advantages of a single-process formation, good anti-compressive and anti-bending performance, and overall integrity [7,8,9,10,11]. Research studies have developed a practical and effective method for overcoming the easy detachment of the honeycomb core from the face sheets of traditional honeycomb panels and have resulted in new types of honeycomb panels with unique mechanical properties. The integrated honeycomb panels that were reported by Chen *et al.* are basalt fiber reinforced epoxy resin composites with a honeycomb wall thickness of approximately 2 mm [9,10,11]. However, in the currently popular honeycomb panels that have aluminum or paper cores, the core wall thickness is less than 0.1 mm. Metallic honeycomb sandwich panels that are composed of thin upper and lower metal panels with an aluminum honeycomb core have many advantages, including being lightweight, having high stiffness, providing noise insulation, and being fire-resistant. These sandwich panels maintain their advantages over time and will be widely used in aviation, high-speed trains, ships, and other fields in the future [12,13,14]. This paper proposes that in the civil construction industry, these panels can be assembled to form a fabricated long-span hollow core roof system, a new spatial structure, by using reliable connectors to combine high-performance aluminum alloy honeycomb plates [15]. This new spatial structure is lightweight, has a high stiffness, and has a low total cost, and these panels can be widely used in several types of long-span structures, such as long-span roofs in single-story industrial buildings, stadiums, and hangars (Figure 1).

Our previous studies (Figure 1c) of high-performance aluminum honeycomb panels in honeycomb panel structural system assembly tests [16,17] showed that honeycomb panels in such structures are primarily under stress and that the buckling of thin honeycomb panels subjected to in-plane loads is one of the key problems in the analysis and design of these new structures. Because the “thin honeycomb panel structural system” is a new spatial structure that is assembled completely from honeycomb panels, it has not been sufficiently studied experimentally or theoretically. Researchers have primarily conducted a series of studies of the buckling of honeycomb paperboard. Ji *et al.* [18] conducted experimental research on the edgewise compressive strength of honeycomb paperboard by measuring and analyzing the effects of factors such as humidity, temperature, and loading rate. Shao *et al.* [19] studied the edgewise compressive strength of honeycomb paperboard with unglued defects using an edgewise compressive test. They recorded and analyzed the deformation and failure patterns of honeycomb paperboard under an edgewise compressive load and determined the edgewise compressive strength of honeycomb paperboard with unglued defects and the rule governing the influence such defects on the edgewise compressive strength. On that basis, Yang *et al.* [20] performed numerical simulations and created a finite element model based on the Tsai–Hill failure criterion. Therefore, this study conducted lateral compressive tests and finite element analyses of honeycomb panels of different lengths and thicknesses. The results revealed the performance and failure characteristics of lateral compressive buckling of the aluminum honeycomb panel structure. In addition, this study proposed a finite element-based method of analysis to provide a basis for engineering applications of high performance aluminum honeycomb panels in the design of long-span structures in the future.

## 2. Results and Discussion

This section discusses the failure mode in the honeycomb panel lateral compressive tests, the finite element analysis stress and strain contour plots and the stress–strain curves. The relationship between the honeycomb panel failure mode and the buckling performance is briefly analyzed and discussed. In addition, the advantages of long-span roof systems and their application prospects are discussed.

### 2.1. Lateral Compressive Failure Mode of the Honeycomb Panels

#### 2.1.1. Failure Mode of the Honeycomb Panels in the Lateral Compressive Tests

**Figure 2:**
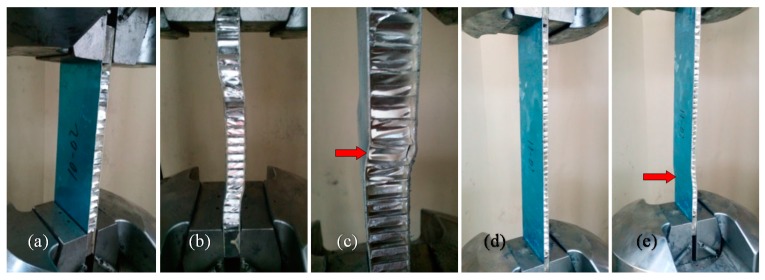
Damage processes in the (**a**–**c**) thick panel and (**d**,**e**) thin panel test pieces; (**a**,**d**) initial loading stage; (**b**,**e**) damaged condition; and (**c**) local magnification of (**b**).

Figure 2 shows the lateral compressive failure process of the thick and thin panels. The upper and lower panels bear the majority of the axial load, regardless of the type of honeycomb panel. The core mainly restricts the face sheet deformation. During the initial loading stage, the panels showed no obvious deformation (Figure 2a,d). The sound of the honeycomb core being flattened was heard as the loading continued; most of the core buckled under the compression of the face sheet (arrows in Figure 2). The overall bending failure of the honeycomb panel then occurred with a muffled bang. The thick honeycomb panel buckled almost entirely; the entire test sample was involved from the face sheet to the collapse of the core by twisting (arrows in Figure 2b,c). However, only a local area of core flattening occurred in the thin honeycomb panel. The face sheet showed slight buckling, but it was not as significant, as it was in the thick type (arrow in Figure 2e).

#### 2.1.2. Stress, Strain and Other Contour Plots of the Finite Element Analysis

**Figure 3:**
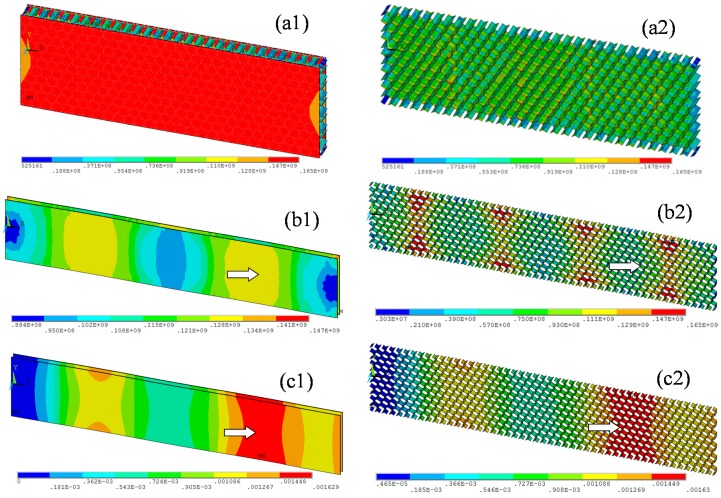
Finite element analysis results of the test pieces. The left column shows the entire panel, and the right column shows the honeycomb core: (**a**) Thick-20; (**b**,**c**) the stress and displacement plots of Thin-10, respectively.

Figure 3 shows the results of the nonlinear analysis of test pieces Thick-20 and Thin-10. Figure 3a shows that most of the honeycomb core did not yield under the lateral compressive load (Figure 3(a2)); the stress was relatively high only at locations that contact the face sheet. However, the honeycomb face sheet almost reached the intensity limit (Figure 3(a1)); that is, the honeycomb panel yielded entirely before lateral compressive buckling occurred. Figure 3b shows that the stress of some of the honeycomb core was too high under the lateral compressive load (wide arrow in Figure 3(b2)) and reached the intensity limit of the aluminum alloy material. This high load caused parts of the core to collapse, which led to a loss of the face sheet’s support function. Although the limit stress of the honeycomb face sheet had not been reached (wide arrow in Figure 3(b1)), failure occurred due to partial buckling near the edge of the honeycomb panel’s structure (wide arrow in Figure 3c). These results show that the failure process is generally consistent with the results of the lateral compressive tests of both the thick and thin honeycomb panels (wide arrows in Figure 2e and Figure 3c). These results are also consistent with those of previous studies; [16,17] the main failure mode of thin honeycomb panels under in-plane load is buckling. Therefore, the buckling of thin honeycomb panels under in-plane loads is a key problem in the analysis and design of such new structures.

### 2.2. Lateral Compressive Displacement Curves for the Honeycomb Panels

**Figure 4:**
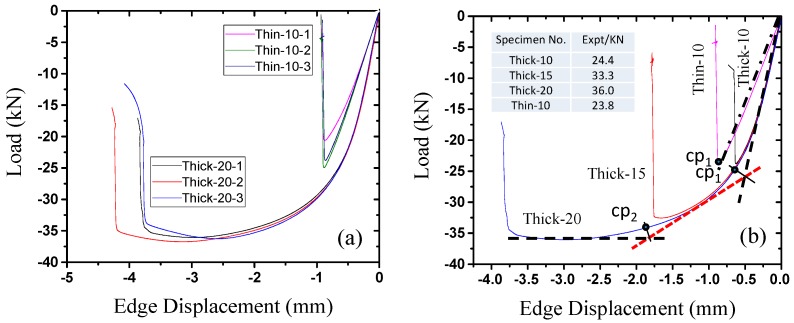
Load–displacement curves of lateral compression test pieces: (**a**) divergence of the internal components of the “thick type” and “thin type”; (**b**) overall results of the test pieces.

Figure 4 shows the load–displacement curves of the test pieces. Figure 4a shows examples of the specimens with the highest thickness-to-length ratio, Thick-20, and the lowest thickness-to-length ratio, Thin-10, which show the divergence of the load–displacement curves of the individual components of each sample. Figure 4b shows the curves of four complete test samples. Figure 4a shows that the divergence between the individual components of each test piece set is small; thus, the test pieces have good stability. This result illustrates the appropriateness and reliability of the test piece preparation process and the mechanical testing method. Clear differences in the compressive stiffness and maximum lateral compressive buckling load can be seen in the load–displacement curves of the thick and thin honeycomb panels (Figure 4b). Specifically, the stiffness of the thick honeycomb panels is significantly greater than that of the thin panels (dashed and dotted lines before cp_1_ in Figure 4c). The load of the thin honeycomb panel is proportional to the edge displacement before failure of the test piece, which indicates that the panel remained in the elastic stage during this process (dotted line in Figure 4b). When the load reached its peak value, part of the core collapsed, and local buckling of the face sheet occurred; the load-bearing capacity of the honeycomb panel decreased rapidly, and the entire test piece experienced instability failure. The thick honeycomb panels showed linear characteristics before the load reached approximately 25 kN. The structure then entered the plastic and reinforcement stage until failure occurred when the load reached a peak value of approximately 35 kN (dashed line in Figure 4b). Figure 4b shows that the stiffnesses of all of the thick specimens were approximately the same even with different thicknesses (dotted line on the right side of Figure 4b). Therefore, the stiffness in the initial elastic stage is mainly determined by the exterior dimensions of the honeycomb panels. However, the panel thickness has a greater influence on the maximum lateral compressive buckling load. Figure 4b shows that, when the panel thickness increased from 10 to 15 mm, the maximum lateral compressive buckling load increased rapidly by approximately 40%. However, when the panel thickness increased from 15 to 20 mm, the maximum lateral compressive buckling load only increased by 10%, which is approximately one-fourth that of the former and approaches a limiting value. We speculate that this limit depends on the dimensions and physical properties of the honeycomb panel.

**Figure 5:**
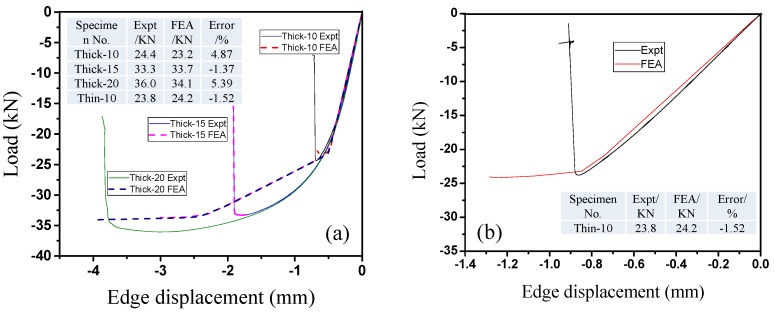
Load–displacement curves from lateral compressive tests and finite element simulations of the honeycomb panels: (**a**) thick test piece; (**b**) thin test piece.

Figure 5 shows the load–displacement curves of the lateral compressive tests of the honeycomb panels and those simulated by finite element models along with the errors. A comparison of the results shows that the finite element simulation results fit the testing results very well. Because a three-segment simplified model was used for the aluminum alloy material during the finite element analysis (Figure 2e), the numerically simulated curve has an obvious yielding point and reinforcement stage; however, the increasing trends of these two load–displacement curves are consistent. Figure 5 shows that the errors between the theoretical and test values are all less than 5.5%, which indicates that the results of the finite element model that was constructed based on the actual components are reliable. This finding lays the theoretical groundwork for using finite element methods in future engineering applications of high-performance aluminum honeycomb panels.

### 2.3. Analysis and Discussion

Unlike the compressive failure of isotropic panels, the failure of honeycomb panels is due to the overall or local buckling failure of the face sheets, which is caused by large shear deformation of the honeycomb core during the loading process (Figure 2). In the thick honeycomb panels, the honeycomb core had sufficient constraints on the face sheet, and the face sheet stress reached its yielding strength; at this time, part of the core collapsed (Figure 2b,c). As loading continued, the face sheets entered the plastic state, and the edge displacement increased rapidly; many of the honeycomb cores lost their constraining effect on the face sheets due to buckling, and face sheet buckling occurred, which led to a rapid decrease in the load-bearing capacity and failure. The theoretical buckling load is lower for the thin honeycomb panels; the internal honeycomb core had already experienced local buckling failure while the face sheets were still in the elastic stage (arrow in Figure 2e).

Regarding the differences and similarities between the load–displacement curves of thick and thin honeycomb panels, in contrast to the clear differences in the anti-compressive stiffness that are shown in Figure 4a, the maximum lateral compressive load of the thin honeycomb panels is very similar to that of the thick panels (Figure 4b); the former is only 5% lower than the latter. Thus, the thickness of the honeycomb core influences the lateral compressive buckling performance of the honeycomb panel; test pieces with the same length but different thicknesses had the same anti-compressive stiffness before buckling. As the thickness of the honeycomb core increased from 10 to 20 mm, the honeycomb panel’s lateral compressive buckling load capacity increased significantly. Moreover, this increase had a larger magnitude from 10 to 15 mm than from 15 to 20 mm. Based on this result, under the testing conditions, increasing the thickness beyond 20 mm would have a minimal effect on the load capacity because the limit would have already been reached. Considering factors such as cost, the thickness of the honeycomb panels should be 15–20 mm. In addition, the anti-compressive stiffnesses of panels with the same thickness but different lengths are different, while the maximum lateral compressive buckling loads are very similar; that is, the longer the honeycomb panel is, the more flexible the structure is, and the more easily overall instability failure occurs. To avoid this type of failure, the design thickness-to-length ratio should be controlled. Figure 4b shows that the lateral compressive buckling performance of a 500-mm-long thick honeycomb panel is slightly less than that of a 300-mm-long thin panel. For construction efficiency, the panel length in the honeycomb panel structure system should be 500 mm.

The new honeycomb panel structure for long-span hollow core roof systems that was developed based on bionic principles has the following basic characteristics: (1) Light self-weight and low production cost. Because the main component of this type of structure is 10–20-mm-thick honeycomb panels and a single panel weighs only 6–7 kg/m^2^, the newly constructed structure has approximately half the self-weight of a regular grid (shell) structure. For a stadium roof design with horizontal dimensions of 60 m × 60 m, although the price of the honeycomb panels is relatively high, the total product cost is not high when the effect of the self-weight is considered [16,17]; (2) Industrialized production and easy and rapid construction. All of the components in the system (*i.e.*, honeycomb panels and connectors) can be produced in a factory. After the panels are transported to the construction site, they can be constructed into unit bodies on the ground or lifted into place after segment assembly; (3) High structural stiffness and good seismic performance. The structural system is a hollow box-like “panel system structure” that is composed of large numbers of lightweight and high strength honeycomb panels. Compared to a “beam system structure” of the same span, the panel structure has better spatial stiffness and seismic performance; (4) Excellent architectural physics performance. The honeycomb panels have intrinsic advantages of sound isolation, heat isolation, and fire resistance; (5) A roof structure that is assembled with honeycomb panels is akin to a trellis-type air-filled “double layered hollow core structure”. These outstanding features can be regarded as the result of an ingenious combination of human wisdom and biological structures. With the continued emergence of super-tall or long-span building structures, this traditional bionic honeycomb panel structure will provide the advantages of light weight and high strength through the suitable choices of materials and structural parameters and contribute to providing mankind with more comfortable living conditions.

## 3. Experiment and Finite Element Analysis Method

### 3.1. Lateral Compressive Tests on Honeycomb Panels

**Figure 6:**
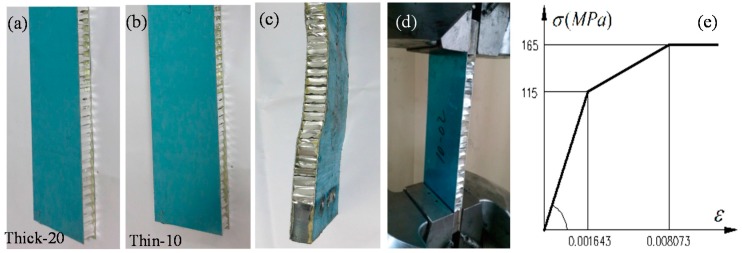
(**a**) The “thick” test piece; (**b**) the “thin” test piece; (**c**) the end reinforcement; (**d**) the test setup; and (**e**) the analytical material constitutive relationship.

To examine the influence of the geometric dimensions of the honeycomb panel structure on the lateral compressive buckling performance, 4 honeycomb panel lateral compressive test pieces with different lengths and thicknesses were designed according to the “honeycomb sandwich structure lateral compressive performance test method” standard [21,22]. All of the test pieces were 100 mm wide, the upper and lower face sheets were 1 mm thick, the sides of the regular hexagonal honeycomb core were 6 mm long, and the core walls were 0.05 mm thick. Three of the 4 test pieces were 300 mm long and had thicknesses of 10, 15, and 20 mm, and the fourth test piece was 500 mm long and 10 mm thick (Figure 6b). For convenience, the three panels with larger thickness-to-length ratios are simply called “thick” test pieces (denoted by specimen numbers Thick-10, -15, and -20, respectively), and the fourth panel is called “thin” (Thin-10). Figure 6a,b show images of the test pieces before the tests. The material that was used for the honeycomb test panels was 3003-H24 type aluminum alloy; an adhesive bonding process was used between the face sheets and the honeycomb core. Because aluminum honeycomb core material is relatively soft, to prevent the collapse of the honeycomb core at the clamped ends during the loading process and increase the local compressive bearing capacity, pieces of iron were placed on the two ends of the test pieces for local reinforcement during the preparation of the test pieces (Figure 6c). The experimental setup and the clamps are shown in Figure 6d. The test used displacement loading with a loading speed of 1 mm/min. The load was applied to the test pieces according to a specified loading mode; uniform and continuous loading were applied until failure, and each type of test piece underwent three tests.

### 3.2. Finite Element Analysis Method

ANSYS software was used to develop the honeycomb panel lateral compressive model for the test samples. The material attributes were as follows: elastic modulus *E* = 70.0 GPa, shear modulus *G* = 27.0 GPa, yield stress σ_0.2_ = 115.0 MPa and limit stress σ_b_ = 165.0 MPa. The simplified constitutive relationship of the specific aluminum alloy is shown in Figure 6e. To construct the finite element model, shell elements were used for the honeycomb core, solid elements were used for the upper and lower face sheets, and interface elements were used in between the face sheets and the core for connections [23,24,25,26,27,28,29,30]. One end of the finite element model was fixed, and the other end was allowed to slide; a unidirectional lateral displacement was applied to the sliding end to exert a load at the edge surface of the honeycomb panel.

## 4. Conclusions

This paper focused on the characteristics of a new honeycomb panel structure system that is mostly in a compressed state and conducted a nonlinear finite element buckling analysis based on an experimental study of the lateral compression of honeycomb panels with different thickness-to-length ratios. The following conclusions were obtained:

1. Face sheet yielding failure occurred in the thick honeycomb panel, which has a lower thickness-to-length ratio. Under the conditions of this experiment, the honeycomb panels with the same planar dimensions but different thicknesses had the same anti-compressive stiffness before buckling, while the lateral compressive buckling load-bearing capacity initially increased rapidly with increasing thickness and eventually approached a limiting value. Under the test conditions in this paper, the increase in load-bearing capacity approached this limit when the thickness of the honeycomb panel exceeded 20 mm. Considering factors such as cost, the honeycomb panels should be 15–20 mm thick.
2. The anti-compressive stiffnesses are different in test pieces with different lengths but the same thickness; however, the maximum lateral compressive buckling loads are very similar. In thin honeycomb panels with high length-to-thickness ratios, overall buckling failure occurred due to local buckling that was induced by the collapse of several honeycomb cores or due to the lack of support from the core to the face sheets; that is, the longer the honeycomb panel is, the more flexible the structure is, and the more likely it is that overall instability failure will occur. To prevent failures of this type, the design length-to-thickness ratio should be controlled.
3. The error between the results of the nonlinear finite element analysis of honeycomb panel models and the actual test results was less than 6%, and the load–displacement curves that were obtained by the two methods were very similar. These results indicate that the finite element analysis model that was developed in this paper has sufficient calculation accuracy and can provide a theoretical basis for the more rational analysis and design of this new type of spatial structure in the future.

## References

[1] Ma Y., Zheng Y., Meng H., Song W., Yao X., Lv H. (2013). Heterogeneous PVA hydrogels with micro-cells of both positive and negative Poisson’s ratios. J. Mech. Behav. Biomed. Mater..

[2] Dirks J.-H., Dürr V. (2011). Biomechanics of the stick insect antenna: Damping properties and structural correlates of the cuticle. J. Mech. Behav. Biomed. Mater..

[3] Koester K.J., Barth H.D., Ritchie R.O. (2011). Effect of aging on the transverse toughness of human cortical bone: Evaluation by R-curves. J. Mech. Behav. Biomed. Mater..

[4] Donius A.E., Liu A., Berglund L.A., Wegst U.G.K. (2014). Superior mechanical performance of highly porous, anisotropic nanocellulose–montmorillonite aerogels prepared by freeze casting. J. Mech. Behav. Biomed. Mater..

[5] Chen J., Gu C., Guo S., Wan C., Wang X., Xie J., Hu X. (2012). Integrated honeycomb technology motivated by the structure of beetle forewings. Mater. Sci. Eng. C.

[6] Chen J., Xie J., Zhu H., Guan S., Wu G., Noori M.N., Guo S. (2012). Integrated honeycomb structure of a beetle forewing and its imitation. Mater. Sci. Eng. C.

[7] Chen J., Xie J., Wu Z., Elbashiry E.M.A., Lu Y. (2015). Review of beetle forewing structures and their biomimetic applications in China: (I) On the structural colors and the vertical and horizontal cross-sectional structures. Mater. Sci. Eng. C.

[8] Chen J., Zu Q., Wu G., Xie J., Tuo W. (2015). Review of beetle forewing structures and their biomimetic applications in China: (II) On the three-dimensional structure, modeling and imitation. Mater. Sci. Eng. C.

[9] Chen J., Wu G. (2013). Beetle forewings: Epitome of the optimal design for lightweight composite materials. Carbohydr. Polym..

[10] Chen J., He C., Gu C., Liu J., Mi C., Guo S. (2014). Compressive and flexural properties of biomimetic integrated honeycomb plates. Mater. Des..

[11] He C., Chen J., Wu Z., Xie J., Zu Q., Lu Y. (2015). Simulated effect on the compressive and shear mechanical properties of bionic integrated honeycomb plates. Mater. Sci. Eng. C.

[12] Bourada M., Tounsi A., Houari M.S.A., Bedia E.A.A. (2011). A new four-variable refined plate theory for thermal buckling analysis of functionally graded sandwich plates. J. Sandw. Struct. Mater..

[13] Szyniszewski S., Smith B.H., Hajjar J.F., Arwade S.R., Schafer B.W. (2012). Local buckling strength of steel foam sandwich panels. Thin-Walled Struct..

[14] Boudjemai A., Amri R., Mankour A., Salem H., Bouanane M.H., Boutchicha D. (2012). Modal analysis and testing of hexagonal honeycomb plates used for satellite structural design. Mater. Des..

[15] Zhao C., Jun M., Yin L., Zhao H. (2008). Assembled Honeycombed Sheet Light Empty Stomach Building and Roof Structure System.

[16] Zhao C., Ma J., Tao J. (2014). Experimental study on load capacity of new fabricated honeycomb panel open-web roof structures. J. Southeast Univ. (Nat. Sci. Ed.).

[17] Tao J. (2012). Experimental Study on Lightweighted Roof Structures Based on Honeycomb Panel in High Performance. Master’ Thesis.

[18] Ji H.-W., Xu J., Li J.-C., Shao W.-Q., Wang H.-W. (2006). Experimental research on the edgewise compressive strength of honeycomb paperboard. Packag. Eng..

[19] Shao W.-Q., Li Y.-M., Meng X.-W., Ji H.-W. (2008). Influence of the unglued defect on edgewise compressive strength of honeycomb paperboard. Packag. Eng..

[20] Yang S., Wu L., Sun Y. (2007). End compression failure of honeycomb sandwich panels containing interfacial debonding. Acta Mater. Compos. Sin..

[21] Standardization Administration of China (2005). Test Method for Edgewise Compressive Properties of Sandwich Constructions.

[22] Standardization Administration of China (1986). The Generals of Test Method for Properties of Adhesive-Bonded Aluminum Honeycomb-Sandwich Structure and Core.

[23] Wang Y., Shi Y., Yuan H., Cheng M. (2011). Compressive buckling of aluminum alloy plates. Eng. J. Wuhan Univ. (Eng. Ed.).

[24] Zhang F., Huang B., Niu W., Cui S. (2000). Buckling and postbuckling analysis of nonlinear composite laminates with delamination. J. Northeast. Univ. (Nat. Sci.).

[25] Zhuang X. (2009). The Buckling and Postbuckling Analysis of Flat Composite Stiffened Panels Loaded in Axial Compression. Master’s Thesis.

[26] Liu C. (2009). Buckling, Post-Buckling and Load-Carrying Capacity Analysis of Stiffened Composite Panels. Master’s Thesis.

[27] Yang N., Shen S. (2003). Finite element analysis for nonlinear buckling of plates and shells. J. Harbin Inst. Technol..

[28] Lou J. (2013). Bending, Buckling and Vibration Properties of Composite Lattice Sandwich Structures. Ph.D. Thesis.

[29] Zhang X. (2013). Research on the Static and Dynamic Buckling of the Composite Honeycomb Structure in the Out-of-Plane Direction. Master’s Thesis.

[30] Yang Y. (2013). Research on Mechanical Properties of Metal Honeycomb Sandwich Plate. Master’s Thesis.

